# Supplementary material for: The Evolution and Characterization of the RNA Interference Pathways in Lophotrochozoa
Source: Genome Biol Evol. 2024 May 7;16(5):evae098. doi: 10.1093/gbe/evae098 (PMC11114477; doi:10.1093/gbe/evae098)
Supplement: evae098_Supplementary_Data [file evae098_supplementary_data.zip › Supplementary_materials.zip!/selected_miRNAs/NoGe_selected_miRNAs/mirdeep_pdf_files/NMRB01002279.1_Notospermus_geniculatus_strain_Ushimado_scaffold2279,_whole_genome_shotgun_sequence_37122.pdf]

Provisional ID	:	NMRB01002279.1_Notospermus_geniculatus_strain_Ushimado_scaffold2279,_whole_genome_shotgun_sequence_37122
Score total	:	88862.4
Score for star read(s)	:	3.9
Score for read counts	:	88854
Score for mfe	:	1.5
Score for randfold	:	0
Score for cons. seed	:	3
Total read count	:	174295
Mature read count	:	174095
Loop read count	:	0
Star read count	:	200

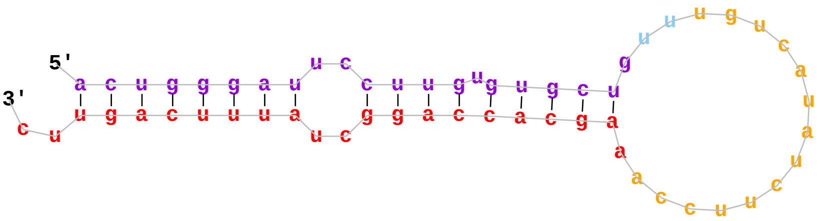

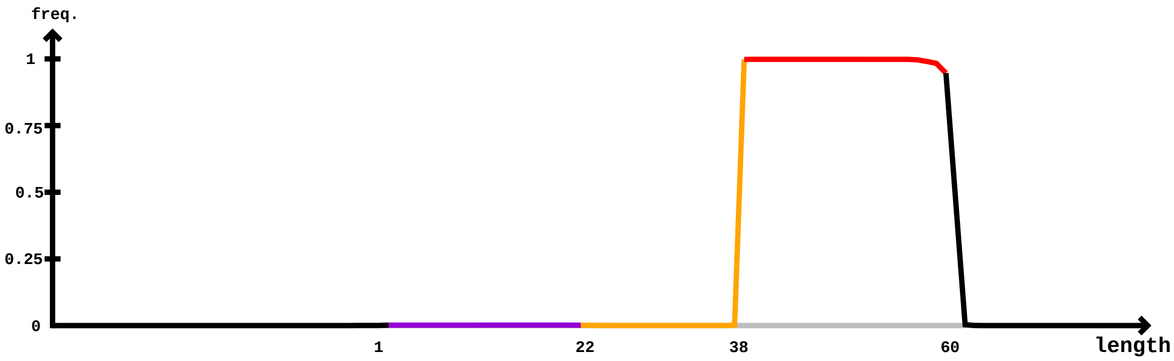

Star Mature

```
5'- agccagaacggagcugcugaaccuauccaguggacugggauuccuugugugcuguuugucauaucuuccaaagcaccaggcuauuucaguucucugguugggagaauuccac  -3'   obs
    agccagaacggagcugcugaaccuauccaguggacugggauuccuugugugcuguuugucauaucuuccaaagcaccaggcuauuucaguucucugguugggagaauuccac         exp
    .........(((....((...((((.((((.((((((((((..((((.(((((..................)))))))))..)))))))))).)))).))))))...)))..       reads    mm    sample
    ..............................uggacugggauuccuugugugcu...........................................................       7        0     Mm3
    ..............................uggacugggauuccuugugugcuU..........................................................       1        1     Mm3
    .................................acugggauuccuugugugcug..........................................................       3        0     Mm3
    .................................acugggauuccuugugugcugu.........................................................       2        0     Mm3
    .................................acugggauuccuugugugcuguu........................................................       8        0     Mm3
    .................................acugggauuccuugugugcuguuu.......................................................       8        0     Mm3
    .................................acugggauuccuugugugcuguuug......................................................       3        0     Mm3
    .................................acugggauuccuugugugcuguuuguc....................................................       2        0     Mm3
    ...............................................ugugcuguuugucauaucuucca..........................................       1        0     Mm3
    ........................................................ugucauaucuuccaaagcaccaggcu..............................       1        0     Mm3
    ..........................................................ucauaucuuccaaagcaccaggc...............................       2        0     Mm3
    ..........................................................ucauaucuuccaaagcaccaggcu..............................       4        0     Mm3
    ..........................................................ucauaucuuccaaagcaccaggcuauuucaguuc....................       1        0     Mm3
    ..........................................................ucauaucuuccaaagcaccaggcuauuucaguucu...................       1        0     Mm3
    .....................................................................aaagcaccaggcuauuucaguu.....................       2        0     Mm3
    .....................................................................aaagcaccaggcuauuucaguuc....................       4        0     Mm3
    .....................................................................Caagcaccaggcuauuucaguuc....................       2        1     Mm3
    ......................................................................aagcaccaggcuauuuca........................       22       0     Mm3
    ......................................................................aagcaccaggcuauuucag.......................       16       0     Mm3
    ......................................................................aagcaccaggcuauuucaA.......................       1        1     Mm3
    ......................................................................aagcaccaggcuauuucagu......................       82       0     Mm3
    ......................................................................aagcaccaggUuauuucagu......................       1        1     Mm3
    ......................................................................aagcaccaggcuauuuAaguu.....................       1        1     Mm3
    ......................................................................aagcacUaggcuauuucaguu.....................       1        1     Mm3
    ......................................................................aagcaccaggcuauuucaguA.....................       1        1     Mm3
    ......................................................................aagcGccaggcuauuucaguu.....................       1        1     Mm3
    ......................................................................aagcaccaggcuauuuUaguu.....................       1        1     Mm3
    ......................................................................Uagcaccaggcuauuucaguu.....................       1        1     Mm3
    ......................................................................aagcaccaggcuauuucaguu.....................       354      0     Mm3
    ......................................................................aagcaccaggcuauuucUguu.....................       4        1     Mm3
    ......................................................................aagcaccaggcuaAuucaguuc....................       1        1     Mm3
    ......................................................................aagcaccaggcuauuucaguAc....................       2        1     Mm3
    ......................................................................Uagcaccaggcuauuucaguuc....................       3        1     Mm3
```

Star **Mature**

```
agccagaacggagcugcugaaccuauccaguggacugggauuccuugugugcuguuugucauaucuuccaaagcaccaggcuauuucaguucucugguugggagaauuccac
......................................................................aagcaccaggcuauuucaguCc....................    2      1    Mm3
......................................................................aagcaccaggcuGuuucaguuc....................    1      1    Mm3
......................................................................aagcaNcaggcuauuucaguuc....................    1      1    Mm3
......................................................................aagcaUcaggcuauuucaguuc....................    2      1    Mm3
......................................................................aagcaccaggcuauuucUguuc....................    14     1    Mm3
......................................................................aagcaccaggcuauuucaUuuc....................    2      1    Mm3
......................................................................aagcaccaggcuauuuGaguuc....................    4      1    Mm3
......................................................................aagcaAcaggcuauuucaguuc....................    1      1    Mm3
......................................................................aagcaccaUgcuauuucaguuc....................    2      1    Mm3
......................................................................aagcaccaggcuauuCcaguuc....................    1      1    Mm3
......................................................................aaAcaccaggcuauuucaguuc....................    3      1    Mm3
......................................................................aagcaccaggcuauuucagGuc....................    1      1    Mm3
......................................................................aagcaccaggcuUuuucaguuc....................    2      1    Mm3
......................................................................aagcaccaggcuauuucaguuc....................    5522   0    Mm3
......................................................................Gagcaccaggcuauuucaguuc....................    1      1    Mm3
......................................................................aagcaccaggcuauuuUaguuc....................    20     1    Mm3
......................................................................aagcaccCggcuauuucaguuc....................    1      1    Mm3
......................................................................aGgcaccaggcuauuucaguuc....................    1      1    Mm3
......................................................................aagcaccaggAuauuucaguuc....................    6      1    Mm3
......................................................................aagcaccaggcuauuucaguuU....................    36     1    Mm3
......................................................................aagcaccUggcuauuucaguuc....................    2      1    Mm3
......................................................................aagcaccaggcuauuucaguuA....................    10     1    Mm3
......................................................................aagcaccaggcuauuucGguuc....................    20     1    Mm3
......................................................................aagcacUaggcuauuucaguuc....................    4      1    Mm3
......................................................................aagcaccaggGuauuucaguuc....................    8      1    Mm3
......................................................................aagcaccGggcuauuucaguuc....................    1      1    Mm3
......................................................................aaUcaccaggcuauuucaguuc....................    2      1    Mm3
......................................................................aagcaccaggUuauuucaguuc....................    4      1    Mm3
......................................................................aagcaccagAcuauuucaguuc....................    2      1    Mm3
......................................................................aCgcaccaggcuauuucaguuc....................    2      1    Mm3
......................................................................Nagcaccaggcuauuucaguuc....................    6      1    Mm3
......................................................................aagcaccaggcuauuucCguuc....................    17     1    Mm3
......................................................................aagcaccaggcuauuuAaguuc....................    25     1    Mm3
......................................................................aagcaccaggcuauuucaguuUu...................    2      1    Mm3
......................................................................aagcaccaggcuauuuUaguucu...................    1      1    Mm3
......................................................................aagUaccaggcuauuucaguucu...................    1      1    Mm3
......................................................................aagcaccaggcuauuuAaguucu...................    1      1    Mm3
......................................................................aagcaccaggcuauuuGaguucu...................    1      1    Mm3
......................................................................aagcaccaggGuauuucaguucu...................    1      1    Mm3
......................................................................aagcaccaggcuauuucaguucA...................    237    1    Mm3
......................................................................aagcaccaggcuauuucaguuAu...................    1      1    Mm3
......................................................................aaCcaccaggcuauuucaguucu...................    1      1    Mm3
......................................................................aagcaNcaggcuauuucaguucu...................    1      1    Mm3
......................................................................aagcaccaggcuauuucaguucu...................    444    0    Mm3
......................................................................aagcaccaggcuauuucaguucG...................    1      1    Mm3
......................................................................aagcaccaggcuauuucGguucu...................    2      1    Mm3
......................................................................aagcacUaggcuauuucaguucu...................    1      1    Mm3
......................................................................aagcaccaggcuauuucCguucu...................    1      1    Mm3
......................................................................aagcaccaggcuauuucUguucu...................    1      1    Mm3
......................................................................aagcaccaggcuauuucaguucC...................    5      1    Mm3
......................................................................aagcaccaggcuauuucaguucuA..................    2      1    Mm3
......................................................................aagcaccaggcuauuucaguucuU..................    23     1    Mm3
......................................................................aagcaccaggcuauuucaguucuUu.................    5      1    Mm3
.......................................................................agcaccaggcuauuucaguu.....................    1      0    Mm3
.......................................................................agcaccaggcuauuucaguuc....................    5      0    Mm3
.......................................................................agcaccaggcuauuucaguucu...................    1      0    Mm3
........................................................................gcaccaggcuauuucaguuc....................    1      0    Mm3

...........agcugcugaaccuauccagugg...............................................................................    1      0    Fm3
..............................uggacugggUuuccuugugugcu...........................................................    1      1    Fm3
..............................uggacugggauuccuugugugcu...........................................................    30     0    Fm3
.................................acugggauuccuugugAgcug..........................................................    1      1    Fm3
.................................acugggauuccuugugugcug..........................................................    98     0    Fm3
.................................acugggauuccuuguguUcug..........................................................    2      1    Fm3
.................................acugggauuccuugugCgcug..........................................................    1      1    Fm3
.................................acugggauuccuugugugcugu.........................................................    9      0    Fm3
.................................acugggauuccuugugugcuguu........................................................    9      0    Fm3
.................................acugggauuccuugugugcuguuu.......................................................    1      0    Fm3
.................................acugggauuccuugugugcuguuug......................................................    4      0    Fm3
.....................................................................aaagcaccaggcuauuucag.......................    2      0    Fm3
```

```
                                 Star                                          Mature
agccagaacggagcugcugaaccuauccaguggacugggauuccuugugugcuguuugucauaucuuccaaagcaccaggcuauuucaguucucugguugggagaauuccac
.....................................................................aaagcaccaggcuauuucaguuc....................    1      0    Fm3
......................................................................aagcaccaggcuauuucU........................    2      1    Fm3
......................................................................aagcaccaggcuauuuca........................    30     0    Fm3
......................................................................aagcaccaggAuauuucag.......................    1      1    Fm3
......................................................................aagcaccGggcuauuucag.......................    1      1    Fm3
......................................................................aagcaccaggcuauuucGg.......................    3      1    Fm3
......................................................................aagcacAaggcuauuucag.......................    1      1    Fm3
......................................................................aagcaccaggcuauuuUag.......................    3      1    Fm3
......................................................................aagcaccaggcuauuucUg.......................    1      1    Fm3
......................................................................aagcaccaggcuauuuAag.......................    4      1    Fm3
......................................................................aagcaccaggcuauuucag.......................    678    0    Fm3
......................................................................aagcaccaggcuauuucaA.......................    3      1    Fm3
......................................................................aagcaccaggcuauuucUgu......................    1      1    Fm3
......................................................................aagcaccaggcuauuucGgu......................    1      1    Fm3
......................................................................aagcaccaggcuauuucagu......................    141    0    Fm3
......................................................................aagcaccaggcuauuucagA......................    1      1    Fm3
......................................................................aagcaccaggcuaCuucagu......................    1      1    Fm3
......................................................................aagcaccaggcuauuucGguu.....................    1      1    Fm3
......................................................................aagcaccaggcuauuucaguu.....................    401    0    Fm3
......................................................................aagcaccaggAuauuucaguu.....................    1      1    Fm3
......................................................................aagcaccaggcuauuucUguu.....................    2      1    Fm3
......................................................................aaUcaccaggcuauuucaguu.....................    1      1    Fm3
......................................................................aagcaccaggcuauuucaguA.....................    2      1    Fm3
......................................................................aagcaccaggcuauuucCguu.....................    1      1    Fm3
......................................................................aagcaccaggcuauuuAaguu.....................    1      1    Fm3
......................................................................aagcaccaggcuUuuucaguu.....................    1      1    Fm3
......................................................................aagcaccaggcuauuuUaguu.....................    4      1    Fm3
......................................................................aagcaccaggcuauuuGaguu.....................    2      1    Fm3
......................................................................aagcUccaggcuauuucaguuc....................    1      1    Fm3
......................................................................aaUcaccaggcuauuucaguuc....................    2      1    Fm3
......................................................................Gagcaccaggcuauuucaguuc....................    1      1    Fm3
......................................................................aagcaUcaggcuauuucaguuc....................    2      1    Fm3
......................................................................aagcaccaUgcuauuucaguuc....................    1      1    Fm3
......................................................................aagcaccaggcuauuuUaguuc....................    6      1    Fm3
......................................................................aagcaccaggGuauuucaguuc....................    5      1    Fm3
......................................................................Uagcaccaggcuauuucaguuc....................    2      1    Fm3
......................................................................aagcaccaggcuauuuAaguuc....................    8      1    Fm3
......................................................................aagcaccaggcuauuucUguuc....................    6      1    Fm3
......................................................................aagcaccaggcuauuCcaguuc....................    1      1    Fm3
......................................................................aagcaccaggcuauuucCguuc....................    4      1    Fm3
......................................................................aagcaccaggcuauuucaguuA....................    7      1    Fm3
......................................................................aagcaccaggcuauCucaguuc....................    3      1    Fm3
......................................................................aagcaccaggAuauuucaguuc....................    5      1    Fm3
......................................................................aagcaccaggcuaAuucaguuc....................    1      1    Fm3
......................................................................Nagcaccaggcuauuucaguuc....................    1      1    Fm3
......................................................................aagcaccaggcuauuucGguuc....................    6      1    Fm3
......................................................................aagGaccaggcuauuucaguuc....................    1      1    Fm3
......................................................................aagcaccaggcuauuucaguuU....................    20     1    Fm3
......................................................................aGgcaccaggcuauuucaguuc....................    1      1    Fm3
......................................................................aagcaccaggUuauuucaguuc....................    7      1    Fm3
......................................................................aagcaccaggcuauuucaguuc....................    2549   0    Fm3
......................................................................aagcaccaggcuauuuGaguuc....................    3      1    Fm3
......................................................................aagcaccaggcuUuuucaguuc....................    1      1    Fm3
......................................................................aagcacUaggcuauuucaguuc....................    1      1    Fm3
......................................................................aagcaccaggcuauuucaguAc....................    2      1    Fm3
......................................................................aagcaccaggcuauuucaguuG....................    1      1    Fm3
......................................................................aagcaccaggcuauuucaguucC...................    7      1    Fm3
......................................................................aagcaccaggcuauuucaguucA...................    119    1    Fm3
......................................................................aagcaccaggcuauuucaguuUu...................    1      1    Fm3
......................................................................aagcaccaggcuauCucaguucu...................    1      1    Fm3
......................................................................aagcaccaggcuauuucaguucu...................    167    0    Fm3
......................................................................aaAcaccaggcuauuucaguucu...................    1      1    Fm3
......................................................................aagcaccaggcuauuucaguucuA..................    1      1    Fm3
......................................................................aagcaccaggcuauuucaguucuU..................    1      1    Fm3
.......................................................................agcaccaggcuauuucaguu.....................    1      0    Fm3
.......................................................................agcaccaggcuauuucaguuc....................    8      0    Fm3
.......................................................................agcaccaggcuauuucaguucu...................    1      0    Fm3

..............................uggacugggaAuccuugugugcu...........................................................    1      1    Mm1
..............................uggacugggauuccuugugugcu...........................................................    12     0    Mm1
```

Star **Mature**

```
agccagaacggagcugcugaaccuauccaguggacugggauuccuugugugcuguuugucauaucuuccaaagcaccaggcuauuucaguucucugguugggagaauuccac
..............................uggacugggauuccuugGgugcu...........................................................   1     1    Mm1
..............................uggacugggauuccuugCgugcu...........................................................   1     1    Mm1
..............................uggacugggauuccuugugugcuU..........................................................   6     1    Mm1
.................................acugggauuccuuguguCcuguuu.......................................................   1     1    Mm1
.................................acugggauuccuuguguAcuguuu.......................................................   1     1    Mm1
.................................acugggauuccuugugugcuguuu.......................................................   15    0    Mm1
.................................acugggauuccuugugugcuguuug......................................................   10    0    Mm1
.................................acugggauuccuugugugcuguuugucauaucuucc...........................................   1     0    Mm1
..........................................................ucauaucuuccaaagcaUcaggcu..............................   1     1    Mm1
..........................................................ucauaucuuccaaagcaccaggcu..............................   4     0    Mm1
..................................................................uccaaagcaccaggcuauuucaguucuc..................   5     0    Mm1
.....................................................................aaagcaccaggcuauuucaguuc....................   10    0    Mm1
......................................................................aagcaccaggcuCuuuca........................   1     1    Mm1
......................................................................aagcaccaggcuauuuca........................   69    0    Mm1
......................................................................aagcaccaggcuauuucU........................   6     1    Mm1
......................................................................aagcaccaggcuauuucag.......................   32    0    Mm1
......................................................................aagcCccaggcuauuucag.......................   1     1    Mm1
......................................................................aagcaccaggcuauuucaU.......................   1     1    Mm1
......................................................................aagcaccaggcuauuucUgu......................   1     1    Mm1
......................................................................aagcaccaggcuauuuGagu......................   2     1    Mm1
......................................................................aagcaccaggcuauuuAagu......................   2     1    Mm1
......................................................................aagcaccaggcuauuucagu......................   154   0    Mm1
......................................................................Nagcaccaggcuauuucaguu.....................   1     1    Mm1
......................................................................aagcaccaggcuauuuUaguu.....................   3     1    Mm1
......................................................................aagAaccaggcuauuucaguu.....................   1     1    Mm1
......................................................................aagcaccaggcuauuuAaguu.....................   3     1    Mm1
......................................................................aagcaccaggGuauuucaguu.....................   1     1    Mm1
......................................................................aagcaccaggcuauuucaguA.....................   7     1    Mm1
......................................................................aagcaccaggcuauuucaguu.....................   722   0    Mm1
......................................................................aagcaccaggUuauuucaguu.....................   2     1    Mm1
......................................................................aagcaccaggcuauuucUguu.....................   2     1    Mm1
......................................................................aagcaccaggcuauuucCguu.....................   7     1    Mm1
......................................................................Uagcaccaggcuauuucaguu.....................   2     1    Mm1
......................................................................aagcaccaggcuauuucaguG.....................   1     1    Mm1
......................................................................aagcaccaggcuaAuucaguu.....................   1     1    Mm1
......................................................................aagcaccaggcuaCuucaguu.....................   1     1    Mm1
......................................................................aagcaccaggcuauuuGaguu.....................   1     1    Mm1
......................................................................aagcaccaggAuauuucaguu.....................   1     1    Mm1
......................................................................aagcaccaggcuauuucUguuc....................   27    1    Mm1
......................................................................aaCcaccaggcuauuucaguuc....................   1     1    Mm1
......................................................................Nagcaccaggcuauuucaguuc....................   9     1    Mm1
......................................................................aagAaccaggcuauuucaguuc....................   2     1    Mm1
......................................................................aagcaccUggcuauuucaguuc....................   1     1    Mm1
......................................................................aagcaccaggcuaCuucaguuc....................   1     1    Mm1
......................................................................aagcaccaggcuauAucaguuc....................   1     1    Mm1
......................................................................aagcaccagCcuauuucaguuc....................   1     1    Mm1
......................................................................aagcaccGggcuauuucaguuc....................   2     1    Mm1
......................................................................aagcaccaggcuaGuucaguuc....................   1     1    Mm1
......................................................................aagcaccaggcuauuuGaguuc....................   15    1    Mm1
......................................................................aagcaccaggcuaAuucaguuc....................   3     1    Mm1
......................................................................aaUcaccaggcuauuucaguuc....................   3     1    Mm1
......................................................................aagcacUaggcuauuucaguuc....................   2     1    Mm1
......................................................................aagcaccaggcuauuucaguuG....................   1     1    Mm1
......................................................................aagcaccaggcuauuCcaguuc....................   1     1    Mm1
......................................................................aagcaccaggcuauuuAaguuc....................   29    1    Mm1
......................................................................aagcaccaggcuauCucaguuc....................   2     1    Mm1
......................................................................aagcaccaggcGauuucaguuc....................   1     1    Mm1
......................................................................aagcaccaggUuauuucaguuc....................   5     1    Mm1
......................................................................aagcaccaggAuauuucaguuc....................   9     1    Mm1
......................................................................aagcaccaggcuauuucCguuc....................   20    1    Mm1
......................................................................aagcCccaggcuauuucaguuc....................   1     1    Mm1
......................................................................aagcaccaggcuauGucaguuc....................   1     1    Mm1
......................................................................Gagcaccaggcuauuucaguuc....................   1     1    Mm1
......................................................................aaAcaccaggcuauuucaguuc....................   1     1    Mm1
......................................................................aagcaccaggcuauuucGguuc....................   19    1    Mm1
......................................................................aagcaccaggcCauuucaguuc....................   1     1    Mm1
......................................................................aagcaccaggcuauuucaUuuc....................   2     1    Mm1
......................................................................aGgcaccaggcuauuucaguuc....................   2     1    Mm1
......................................................................aagcaccaggcuauuuUaguuc....................   20    1    Mm1
......................................................................aagcaccaggGuauuucaguuc....................   6     1    Mm1
```

```
                                 Star                                 Mature
agccagaacggagcugcugaaccuauccaguggacugggauuccuugugugcuguuugucauaucuuccaaagcaccaggcuauuucaguucucugguugggagaauuccac
......................................................................aagcaccaggcuauuucaguAc....................   2      1    Mm1
......................................................................aagcacAaggcuauuucaguuc....................   1      1    Mm1
......................................................................aagcaccaggcuauuucaguuc....................   7960   0    Mm1
......................................................................aUgcaccaggcuauuucaguuc....................   2      1    Mm1
......................................................................Uagcaccaggcuauuucaguuc....................   7      1    Mm1
......................................................................aagcaccaggcuauuucaguU.....................   19     1    Mm1
......................................................................aagcGccaggcuauuucaguuc....................   2      1    Mm1
......................................................................aagGaccaggcuauuucaguuc....................   2      1    Mm1
......................................................................aagcaNcaggcuauuucaguuc....................   2      1    Mm1
......................................................................aagcaccagUcuauuucaguuc....................   2      1    Mm1
......................................................................aagcaccaggcuauuucGguucu...................   1      1    Mm1
......................................................................Cagcaccaggcuauuucaguucu...................   1      1    Mm1
......................................................................aagcaccaggAuauuucaguucu...................   1      1    Mm1
......................................................................aagcaccaggUuauuucaguucu...................   1      1    Mm1
......................................................................aagcaccaggcuauuuGaguucu...................   2      1    Mm1
......................................................................aagcaccaggcuauuucUguucu...................   1      1    Mm1
......................................................................aagcaccaggcuauuuUaguucu...................   1      1    Mm1
......................................................................aGgcaccaggcuauuucaguucu...................   1      1    Mm1
......................................................................aagcaccaggcuauuucaguucu...................   394    0    Mm1
......................................................................aagcaccaggcuauuucaguucC...................   34     1    Mm1
......................................................................Nagcaccaggcuauuucaguucu...................   1      1    Mm1
......................................................................aagcaccaggcuauuucaguucG...................   10     1    Mm1
......................................................................aagcaccaggcuauuucaguucA...................   455    1    Mm1
......................................................................aagcaccaggcuauuuAaguucu...................   1      1    Mm1
......................................................................aagcaccaggcuauuucaguucuU..................   9      1    Mm1
......................................................................aagcaccaggcuauuucaguucAc..................   2      1    Mm1
......................................................................aagcaccaggcuauuucaguucuUu.................   1      1    Mm1
.......................................................................agcaccaggcuauuucaguu.....................   7      0    Mm1
.......................................................................agcaccaggcuauuucaguuc....................   11     0    Mm1

..............................uggacugggauuccuugugugc............................................................   1      0    Mm2
..............................uggacugggauuccuugugugcu...........................................................   4      0    Mm2
..............................uggacugggauuccuugugugcG...........................................................   1      1    Mm2
.................................acugggauuccuugugugcug..........................................................   3      0    Mm2
.................................acugggauuccuugugugcuguu........................................................   5      0    Mm2
.................................acugggauuccuuguguAcuguuu.......................................................   1      1    Mm2
.................................acugggauuccuugugugcuguuu.......................................................   1      0    Mm2
.................................acugggauuccuugugugcuguuug......................................................   3      0    Mm2
.................................acugggauuccuugugugcuguuugA.....................................................   2      1    Mm2
.................................acugggauuccuugugugcuguuugucau..................................................   1      0    Mm2
.................................acugggauuccuugugugcuguuugucauaucuucca..........................................   1      0    Mm2
...........................................cuugugugcuguuugucauaucuucca..........................................   1      0    Mm2
..........................................................ucauaucuuccaaagcaccaggc...............................   4      0    Mm2
..........................................................ucauaucuuccaaagcaccaggcu..............................   6      0    Mm2
..........................................................ucauaucuuccaaagcaccaggcuauuucaguucu...................   1      0    Mm2
..........................................................ucauaucuuccaaagcaccaggcuauuucGguucu...................   1      1    Mm2
.....................................................................aaagcaccaggcuauuuc.........................   1      0    Mm2
.....................................................................aaagcaccaggcuauuucagu......................   1      0    Mm2
.....................................................................aaagcaccaggcuauuucaguu.....................   2      0    Mm2
.....................................................................aaagcaccaggcuauuucaguuc....................   50     0    Mm2
.....................................................................aaagcaccagAcuauuucaguuc....................   1      1    Mm2
.....................................................................aaagcaccaggcuauuucaguuU....................   2      1    Mm2
.....................................................................Caagcaccaggcuauuucaguuc....................   12     1    Mm2
.....................................................................aaagcaccaggcuauuucaguucu...................   10     0    Mm2
......................................................................aagcaccaggcuauuucG........................   1      1    Mm2
......................................................................aagcaccaggcuauuuUa........................   2      1    Mm2
......................................................................aagcaccaggcuauuucU........................   2      1    Mm2
......................................................................aagcaccaggcuauuuca........................   47     0    Mm2
......................................................................aagcaccaggcuauuucaU.......................   1      1    Mm2
......................................................................aagcaccaggcuauuuAag.......................   1      1    Mm2
......................................................................aagcaccaggcuauuucag.......................   104    0    Mm2
......................................................................aagcaccaggcuauuucaA.......................   4      1    Mm2
......................................................................aagcacUaggcuauuucag.......................   1      1    Mm2
......................................................................aagcaccaggcuauuucagA......................   1      1    Mm2
......................................................................aagcaccaggcuauuucCgu......................   4      1    Mm2
......................................................................aagcaccaggcuauuucagu......................   196    0    Mm2
......................................................................aagcaAcaggcuauuucagu......................   1      1    Mm2
......................................................................aagcaccaggcuauuucaguu.....................   1398   0    Mm2
......................................................................aagcaccaggcuauuCcaguu.....................   1      1    Mm2
......................................................................aGgcaccaggcuauuucaguu.....................   1      1    Mm2
```

Star Mature

```
agccagaacggagcugcugaaccuauccaguggacugggauuccuugugugcuguuugucauaucuuccaaagcaccaggcuauuucaguucucugguugggagaauuccac
......................................................................aagcaccaggcuauuucGguu.....................  3      1  Mm2
......................................................................aagcaccaggcuauuuAaguu.....................  6      1  Mm2
......................................................................aagcaccaggGuauuucaguu.....................  1      1  Mm2
......................................................................aagcaccaggcuauCucaguu.....................  1      1  Mm2
......................................................................aagcaccaggAuauuucaguu.....................  2      1  Mm2
......................................................................aagcaccaggcuauuucaguA.....................  10     1  Mm2
......................................................................aagcaccaggcuauuuUaguu.....................  3      1  Mm2
......................................................................aagcaccaggcuauuucaguC.....................  1      1  Mm2
......................................................................aagUaccaggcuauuucaguu.....................  1      1  Mm2
......................................................................aagcaUcaggcuauuucaguu.....................  2      1  Mm2
......................................................................aagcaccaggcuauuucUguu.....................  8      1  Mm2
......................................................................aagcacUaggcuauuucaguu.....................  1      1  Mm2
......................................................................aagcaccaggcuauuuGaguu.....................  3      1  Mm2
......................................................................aagcaccaggcuauuucCguu.....................  5      1  Mm2
......................................................................aagcUccaggcuauuucaguu.....................  1      1  Mm2
......................................................................aagcaccaggcuauuucaguG.....................  1      1  Mm2
......................................................................aaCcaccaggcuauuucaguu.....................  1      1  Mm2
......................................................................aagcaccaggcuauuucagCu.....................  1      1  Mm2
......................................................................aagcaccaggcuauuAcaguuc....................  7      1  Mm2
......................................................................aagcaccaCgcuauuucaguuc....................  1      1  Mm2
......................................................................Gagcaccaggcuauuucaguuc....................  9      1  Mm2
......................................................................Nagcaccaggcuauuucaguuc....................  22     1  Mm2
......................................................................aagcaccaggAuauuucaguuc....................  94     1  Mm2
......................................................................aagcacAaggcuauuucaguuc....................  4      1  Mm2
......................................................................aagcaccaggcuauGucaguuc....................  1      1  Mm2
......................................................................aagcaccaggcuaCuucaguuc....................  7      1  Mm2
......................................................................aagcaGcaggcuauuucaguuc....................  2      1  Mm2
......................................................................aagcaccaggcAauuucaguuc....................  2      1  Mm2
......................................................................aagcaccaggGuauuucaguuc....................  38     1  Mm2
......................................................................aagcUccaggcuauuucaguuc....................  2      1  Mm2
......................................................................aagcacNaggcuauuucaguuc....................  8      1  Mm2
......................................................................aagcaccaAgcuauuucaguuc....................  4      1  Mm2
......................................................................aCgcaccaggcuauuucaguuc....................  2      1  Mm2
......................................................................aagcaccaggcuauCucaguuc....................  3      1  Mm2
......................................................................aagcaccaggcuauuucaguAc....................  4      1  Mm2
......................................................................aagcaccaggcuauuCcaguuc....................  9      1  Mm2
......................................................................aagcaccaggcuauuucagCuc....................  6      1  Mm2
......................................................................aagcaccaggcuauuucaguuG...................  1      1  Mm2
......................................................................aagcaccagAcuauuucaguuc....................  4      1  Mm2
......................................................................aagcaccaggcuCuuucaguuc....................  2      1  Mm2
......................................................................aagcaAcaggcuauuucaguuc....................  2      1  Mm2
......................................................................aagcaccGggcuauuucaguuc....................  8      1  Mm2
......................................................................aagcaccUggcuauuucaguuc....................  14     1  Mm2
......................................................................Cagcaccaggcuauuucaguuc....................  3      1  Mm2
......................................................................aagcGccaggcuauuucaguuc....................  3      1  Mm2
......................................................................aagcaccaggcuaAuucaguuc....................  14     1  Mm2
......................................................................aagcaccaggcuauuucaguGc....................  2      1  Mm2
......................................................................aagcaccagUcuauuucaguuc....................  3      1  Mm2
......................................................................aagcacGaggcuauuucaguuc....................  4      1  Mm2
......................................................................aagcaccaggcuauuucaAuuc....................  4      1  Mm2
......................................................................aagcaccaggcuauuucUguuc....................  138    1  Mm2
......................................................................aagcaccaggcCauuucaguuc....................  4      1  Mm2
......................................................................aagcaccaggcuauuucagAuc....................  3      1  Mm2
......................................................................aagcaccaggcuauuucagGuc....................  2      1  Mm2
......................................................................aagcaccagCcuauuucaguuc....................  2      1  Mm2
......................................................................aagcaccaggcuUuuucaguuc....................  8      1  Mm2
......................................................................aagcaccaggcuauuucaguuc....................  43690  0  Mm2
......................................................................aagcaccaggcuauuucaguuU...................  203    1  Mm2
......................................................................aagcaccaggcuauAucaguuc....................  2      1  Mm2
......................................................................aagcaccaggcGauuucaguuc....................  3      1  Mm2
......................................................................aagcacUaggcuauuucaguuc....................  12     1  Mm2
......................................................................aUgcaccaggcuauuucaguuc....................  7      1  Mm2
......................................................................aagcaUcaggcuauuucaguuc....................  12     1  Mm2
......................................................................aagcaccaggcuaGuucaguuc....................  4      1  Mm2
......................................................................aagcCccaggcuauuucaguuc....................  2      1  Mm2
......................................................................aaAcaccaggcuauuucaguuc....................  16     1  Mm2
......................................................................aGgcaccaggcuauuucaguuc....................  7      1  Mm2
......................................................................aagcaccaggcuauuuAaguuc....................  191    1  Mm2
......................................................................aagcaccaUgcuauuucaguuc....................  8      1  Mm2
......................................................................aagcaNcaggcuauuucaguuc....................  11     1  Mm2
```

Star **Mature**

```
agccagaacggagcugcugaaccuauccaguggacugggauuccuugugugcuguuugucauaucuuccaaagcaccaggcuauuucaguucucugguugggagaauuccac
......................................................................aagcaccaggUuauuucaguuc....................   30     1   Mm2
......................................................................aagcaccaggcuauuucCguuc....................   133    1   Mm2
......................................................................aagcaccCggcuauuucaguuc....................   4      1   Mm2
......................................................................aagcaccaggcuauuuGaguuc....................   60     1   Mm2
......................................................................Uagcaccaggcuauuucaguuc....................   15     1   Mm2
......................................................................aagAaccaggcuauuucaguuc....................   5      1   Mm2
......................................................................aaUcaccaggcuauuucaguuc....................   17     1   Mm2
......................................................................aagcaccaggcuauuucGguuc....................   129    1   Mm2
......................................................................aagcaccaggcuauuucaguCc....................   8      1   Mm2
......................................................................aagcaccaggcuauuucaguuA....................   14     1   Mm2
......................................................................aagcaccaggcuauuucaUuuc....................   6      1   Mm2
......................................................................aaCcaccaggcuauuucaguuc....................   3      1   Mm2
......................................................................aagcaccaggcuauuuUaguuc....................   114    1   Mm2
......................................................................aagUaccaggcuauuucaguuc....................   4      1   Mm2
......................................................................aagcaccaggcuGuuucaguuc....................   5      1   Mm2
......................................................................aaCcaccaggcuauuucaguucu...................   1      1   Mm2
......................................................................aagcaccaggcuauuucCguucu...................   7      1   Mm2
......................................................................aagcaccaggcuauuucaguuAu...................   1      1   Mm2
......................................................................aagcaccaggcuauCucaguucu...................   1      1   Mm2
......................................................................aagcaccaggUuauuucaguucu...................   5      1   Mm2
......................................................................aagcaccGggcuauuucaguucu...................   1      1   Mm2
......................................................................aagcaccaggcuauuucaguucA...................   1549   1   Mm2
......................................................................aagcaccaggcuauuucGguucu...................   7      1   Mm2
......................................................................aagcaccaggcuaCuucaguucu...................   1      1   Mm2
......................................................................aagcaccaggcuauuucaguucu...................   2107   0   Mm2
......................................................................aagcaccaUgcuauuucaguucu...................   1      1   Mm2
......................................................................aagcaccaggcuauuCcaguucu...................   1      1   Mm2
......................................................................aagcaccaggcuauuucaguucG...................   15     1   Mm2
......................................................................aagcaccaggcuauuucUguucu...................   8      1   Mm2
......................................................................aagcaNcaggcuauuucaguucu...................   1      1   Mm2
......................................................................aagcaccaggcuauuucaguucC...................   93     1   Mm2
......................................................................aagcaccagUcuauuucaguucu...................   1      1   Mm2
......................................................................aagcaccaggcuauuuAaguucu...................   5      1   Mm2
......................................................................aagcaccaggGuauuucaguucu...................   2      1   Mm2
......................................................................aagcaccaggAuauuucaguucu...................   3      1   Mm2
......................................................................aagcaccaggcuauuuUaguucu...................   11     1   Mm2
......................................................................aagcaccaggcuauuucaguuUu...................   10     1   Mm2
......................................................................aagcGccaggcuauuucaguucu...................   1      1   Mm2
......................................................................aGgcaccaggcuauuucaguucu...................   1      1   Mm2
......................................................................aagcaccaggcuauuucaguucuA..................   3      1   Mm2
......................................................................aagcaccaggcuauuucaguucuU..................   76     1   Mm2
......................................................................aagcaccaggcuauuucaguucAc..................   1      1   Mm2
......................................................................aagcaccaggcuauuucaguucuUu.................   16     1   Mm2
.......................................................................agcaccaggcuauuucagu......................   1      0   Mm2
.......................................................................agcaccaggcuauuucaguuc....................   16     0   Mm2
.......................................................................agcaccaggcuauuucaguucu...................   2      0   Mm2

.....................................................................aaagcaccaggcuauuucaguu.....................   7      0   Fm2
.....................................................................aaagcaccaggcuauuuGaguu.....................   1      1   Fm2
.....................................................................aaagcaccaggcuauuucaguuc....................   33     0   Fm2
.....................................................................Caagcaccaggcuauuucaguuc....................   7      1   Fm2
......................................................................aagcaccaggcuauuuca........................   18     0   Fm2
......................................................................aagcaccaggcuauuuGag.......................   1      1   Fm2
......................................................................aagcaccaggcuauuucag.......................   33     0   Fm2
......................................................................aagcaccaggGuauuucag.......................   1      1   Fm2
......................................................................aagcaccaggcuauuuAag.......................   1      1   Fm2
......................................................................aagcaccaggcuauuuAagu......................   1      1   Fm2
......................................................................aagcaccaggcuauuucagu......................   155    0   Fm2
......................................................................aagcaccaggcuauuucCgu......................   1      1   Fm2
......................................................................aagcaccaggcuauuucGgu......................   1      1   Fm2
......................................................................aagcaccagUcuauuucagu......................   1      1   Fm2
......................................................................Nagcaccaggcuauuucagu......................   1      1   Fm2
......................................................................aagcaccaggcuauuucUgu......................   1      1   Fm2
......................................................................aagcaccaggcuauuucUguu.....................   3      1   Fm2
......................................................................aagcaccaggcuauuucaguu.....................   680    0   Fm2
......................................................................aagcaccaggAuauuucaguu.....................   1      1   Fm2
......................................................................Uagcaccaggcuauuucaguu.....................   1      1   Fm2
......................................................................aagcaccaggcuauuuGaguu.....................   1      1   Fm2
......................................................................aagcaccaggcuauuucGguu.....................   1      1   Fm2
......................................................................aGgcaccaggcuauuucaguu.....................   1      1   Fm2
```

```
                                 Star                                   Mature
agccagaacggagcugcugaaccuauccaguggacugggauuccuugugugcuguuugucauaucuuccaaagcaccaggcuauuucaguucucugguugggagaauuccac
......................................................................aagcaccaggcuauuuUaguu.....................   3       1   Fm2
......................................................................aagcaccaggGuauuucaguu.....................   1       1   Fm2
......................................................................aagcaccagCcuauuucaguu.....................   1       1   Fm2
......................................................................aagcaccaggcuauuucCguu.....................   1       1   Fm2
......................................................................aagcaccaggcuauuuAaguu.....................   3       1   Fm2
......................................................................aagcacGaggcuauuucaguuc....................   2       1   Fm2
......................................................................aagcaccaggcuauuucCguuc....................   72      1   Fm2
......................................................................aagcaccaggcuGuuucaguuc....................   3       1   Fm2
......................................................................aagcaccaggcuauuucaguuc....................   23857   0   Fm2
......................................................................aagcaccCggcuauuucaguuc....................   1       1   Fm2
......................................................................aagcaccaggcuauuAcaguuc....................   3       1   Fm2
......................................................................aagcacAaggcuauuucaguuc....................   5       1   Fm2
......................................................................aagAaccaggcuauuucaguuc....................   6       1   Fm2
......................................................................aagcaccaggcuaGuucaguuc....................   2       1   Fm2
......................................................................Uagcaccaggcuauuucaguuc....................   11      1   Fm2
......................................................................aagcaccaggcuauuucaguuU....................   103     1   Fm2
......................................................................aaCcaccaggcuauuucaguuc....................   3       1   Fm2
......................................................................aagcCccaggcuauuucaguuc....................   3       1   Fm2
......................................................................aagcaccaggcuauuucagAuc....................   1       1   Fm2
......................................................................aagcaNcaggcuauuucaguuc....................   6       1   Fm2
......................................................................aCgcaccaggcuauuucaguuc....................   5       1   Fm2
......................................................................aagcaccaggcuaCuucaguuc....................   8       1   Fm2
......................................................................aagcaccaggUuauuucaguuc....................   21      1   Fm2
......................................................................aagcaccaggcuauuuUaguuc....................   58      1   Fm2
......................................................................aagcaccUggcuauuucaguuc....................   5       1   Fm2
......................................................................aNgcaccaggcuauuucaguuc....................   1       1   Fm2
......................................................................aagcGccaggcuauuucaguuc....................   6       1   Fm2
......................................................................aagcaccaggcuauuuGaguuc....................   29      1   Fm2
......................................................................aagcaccaggcuauuuAaguuc....................   110     1   Fm2
......................................................................aagcaccagCcuauuucaguuc....................   2       1   Fm2
......................................................................aagcaAcaggcuauuucaguuc....................   1       1   Fm2
......................................................................aGgcaccaggcuauuucaguuc....................   4       1   Fm2
......................................................................aaUcaccaggcuauuucaguuc....................   8       1   Fm2
......................................................................aagcaccaggcuauuucaguCc....................   1       1   Fm2
......................................................................aagGaccaggcuauuucaguuc....................   1       1   Fm2
......................................................................aagcaccGggcuauuucaguuc....................   3       1   Fm2
......................................................................aagcacUaggcuauuucaguuc....................   4       1   Fm2
......................................................................aagcaccaggcuauAucaguuc....................   2       1   Fm2
......................................................................aagcacNaggcuauuucaguuc....................   2       1   Fm2
......................................................................aaAcaccaggcuauuucaguuc....................   8       1   Fm2
......................................................................aagcaccagUcuauuucaguuc....................   4       1   Fm2
......................................................................Cagcaccaggcuauuucaguuc....................   2       1   Fm2
......................................................................aagcaccaggcuauuucaUuuc....................   5       1   Fm2
......................................................................aagcaccagAcuauuucaguuc....................   3       1   Fm2
......................................................................aagcaccaggcuauuGcaguuc....................   1       1   Fm2
......................................................................aagcaUcaggcuauuucaguuc....................   6       1   Fm2
......................................................................aUgcaccaggcuauuucaguuc....................   1       1   Fm2
......................................................................aagUaccaggcuauuucaguuc....................   1       1   Fm2
......................................................................aagcaccaggcuUuuucaguuc....................   6       1   Fm2
......................................................................aagcaccaggcuauuCcaguuc....................   7       1   Fm2
......................................................................aagcUccaggcuauuucaguuc....................   5       1   Fm2
......................................................................aagcaGcaggcuauuucaguuc....................   1       1   Fm2
......................................................................aaNcaccaggcuauuucaguuc....................   1       1   Fm2
......................................................................aagcaccaAgcuauuucaguuc....................   1       1   Fm2
......................................................................aagcaccaggcCauuucaguuc....................   2       1   Fm2
......................................................................aagcaccaUgcuauuucaguuc....................   10      1   Fm2
......................................................................Gagcaccaggcuauuucaguuc....................   2       1   Fm2
......................................................................Nagcaccaggcuauuucaguuc....................   12      1   Fm2
......................................................................aagcaccaggcAauuucaguuc....................   2       1   Fm2
......................................................................aagcaccaggcuauuucaguuA....................   25      1   Fm2
......................................................................aagcaccaggcuCuuucaguuc....................   1       1   Fm2
......................................................................aagcaccaggcuauuucGguuc....................   94      1   Fm2
......................................................................aagcaccaggcuauuucUguuc....................   74      1   Fm2
......................................................................aagcaccaggAuauuucaguuc....................   44      1   Fm2
......................................................................aagcaccaggGuauuucaguuc....................   28      1   Fm2
......................................................................aagcaccaggcuaAuucaguuc....................   9       1   Fm2
......................................................................aagcaccaggcuauuucCguucu...................   6       1   Fm2
......................................................................aagcaccaggcuauuucGguucu...................   4       1   Fm2
......................................................................aagcaccaggcuauuucaguucG...................   9       1   Fm2
......................................................................aagcaccaggcuauuucaguucC...................   116     1   Fm2
```

Star   Mature

agccagaacggagcugcugaaccuauccaguggacugggauuccuugugugcuguuugucauaucuuccaaagcaccaggcuauuucaguucucugguugggagaauuccac			
....aagcaAcaggcuauuucaguucu...................	1	1	Fm2
....aagcaccaggcuauuucUguucu...................	5	1	Fm2
....aagcaccaggcuaAuucaguucu...................	1	1	Fm2
....aagcaccaggcuGuuucaguucu...................	1	1	Fm2
....aagcaccaggUuauuucaguucu...................	1	1	Fm2
....aagcaUcaggcuauuucaguucu...................	1	1	Fm2
....aagcaccaggcuauuuAaguucu...................	9	1	Fm2
....aagcaccaggcuauuucaguucA...................	1347	1	Fm2
....aagcaccaggGuauuucaguucu...................	5	1	Fm2
....Uagcaccaggcuauuucaguucu...................	2	1	Fm2
....aagcaccaggcuauuucaUuucu...................	1	1	Fm2
....aaUcaccaggcuauuucaguucu...................	2	1	Fm2
....aagcaccaggcuauuuUaguucu...................	2	1	Fm2
....aagcaccaggcuauuucaguucu...................	2071	0	Fm2
....aagcaccaUgcuauuucaguucu...................	1	1	Fm2
....aagcaccaggAuauuucaguucu...................	6	1	Fm2
....aagcaccaggcuauuuGaguucu...................	2	1	Fm2
....aagcaccaggcuauuucaguucuU..................	118	1	Fm2
....aagcaccaggcuauuucaguucuUu.................	22	1	Fm2
....agcaccaggcuauuucaguuc....................	8	0	Fm2
..............................uggacugggauuccuugugugcu...	1	0	Fm1
.................................acugggauuccuugugugcug..	3	0	Fm1
.................................acugggauuccuugugugcGg..	1	1	Fm1
.................................acugggauuccuugugugcuguuu...	3	0	Fm1
....cuugugugcuguuugucauaucuucca..	4	0	Fm1
....ucauaucuuccaaagcaccaggcu..............................	1	0	Fm1
....aaagcaccaggcuauuuAagu......................	1	1	Fm1
....aaagcaccaggcuauuucagu......................	1	0	Fm1
....aaagcacGaggcuauuucaguu.....................	1	1	Fm1
....aaagcaccaggcuauuucaguu.....................	19	0	Fm1
....Caagcaccaggcuauuucaguu.....................	4	1	Fm1
....aaagcaccaggcuauuucaguG.....................	1	1	Fm1
....aaagcaccaggcuauuucaguuc....................	67	0	Fm1
....Caagcaccaggcuauuucaguuc....................	18	1	Fm1
....aaagcaccaggcuauuuAaguuc....................	1	1	Fm1
....Caagcaccaggcuauuucaguucu...................	5	1	Fm1
....aagcaccaggcuauuuca........................	104	0	Fm1
....aagcaccaggcuauuuAa........................	2	1	Fm1
....aagcaccaggcuauuucag.......................	137	0	Fm1
....aagcaccaggcuauuucaU.......................	2	1	Fm1
....aagcaccaggcuauuucUg.......................	1	1	Fm1
....aagcaccaggcuauuucaA.......................	1	1	Fm1
....aagcaccaggcuauuuUag.......................	1	1	Fm1
....aGgcaccaggcuauuucag.......................	1	1	Fm1
....Uagcaccaggcuauuucagu......................	1	1	Fm1
....aagcaccaggcuauuuAagu......................	1	1	Fm1
....Nagcaccaggcuauuucagu......................	1	1	Fm1
....aagcaccaggcuauuucUgu......................	1	1	Fm1
....aUgcaccaggcuauuucagu......................	1	1	Fm1
....aagcaccaggcuauuucagu......................	503	0	Fm1
....aagcaccaggGuauuucagu......................	1	1	Fm1
....aagcaccaggcuauuucCgu......................	1	1	Fm1
....aagcaccaggcuauuucaUu......................	6	1	Fm1
....aagcaccaggcuauuucagA......................	6	1	Fm1
....aagcaccaggcuauuucagC......................	1	1	Fm1
....aagcaccaggcuauuucGgu......................	2	1	Fm1
....aagcaccaggcuauuuGagu......................	1	1	Fm1
....aagcaccaggcuauuuUagu......................	2	1	Fm1
....aagUaccaggcuauuucaguu.....................	1	1	Fm1
....aagcaccGggcuauuucaguu.....................	1	1	Fm1
....aagcaccaggcuauuucagCu.....................	1	1	Fm1
....aagcaccaggAuauuucaguu.....................	7	1	Fm1
....aagcaccaggcuauuucaguA.....................	17	1	Fm1
....aagcaccaggcuauuuUaguu.....................	6	1	Fm1
....aaUcaccaggcuauuucaguu.....................	1	1	Fm1
....aagcaccaggcuauuucaguG.....................	3	1	Fm1
....aagcaccaggcuauuucUguu.....................	4	1	Fm1
....aagcaccaggcuauuucGguu.....................	9	1	Fm1
....aagcaccaggcuauuucaguC.....................	1	1	Fm1

```
                                 Star                                   Mature
agccagaacggagcugcugaaccuauccaguggacugggauuccuugugugcuguuugucauaucuuccaaagcaccaggcuauuucaguucucugguugggagaauuccac
......................................................................aGgcaccaggcuauuucaguu.....................  1      1   Fm1
......................................................................aagcaccaggGuauuucaguu.....................  2      1   Fm1
......................................................................aagcGccaggcuauuucaguu.....................  2      1   Fm1
......................................................................aagcaccaggcuauuuAaguu.....................  10     1   Fm1
......................................................................aagcaccaggcAauuucaguu.....................  1      1   Fm1
......................................................................aaCcaccaggcuauuucaguu.....................  2      1   Fm1
......................................................................aagcaccaggcuauuucaguu.....................  2501   0   Fm1
......................................................................aagcaccaggcuauuucCguu.....................  10     1   Fm1
......................................................................aagcaccaAgcuauuucaguu.....................  1      1   Fm1
......................................................................aagcaccaggcuauuAcaguu.....................  1      1   Fm1
......................................................................aagcaccaggUuauuucaguu.....................  5      1   Fm1
......................................................................aagcaccaggcuaAuucaguu.....................  1      1   Fm1
......................................................................aagcaccaggcuauuCcaguu.....................  2      1   Fm1
......................................................................aagcaccaggcuauuuGaguu.....................  5      1   Fm1
......................................................................aagcUccaggcuauuucaguu.....................  1      1   Fm1
......................................................................aNgcaccaggcuauuucaguu.....................  1      1   Fm1
......................................................................aagcacGaggcuauuucaguu.....................  1      1   Fm1
......................................................................aagcaccaggcCauuucaguu.....................  1      1   Fm1
......................................................................aagcacUaggcuauuucaguu.....................  1      1   Fm1
......................................................................aagcaccaggcuauCucaguuc....................  8      1   Fm1
......................................................................aagcacAaggcuauuucaguuc....................  4      1   Fm1
......................................................................aaNcaccaggcuauuucaguuc....................  1      1   Fm1
......................................................................aagcaccaggcuauuucaguuG....................  6      1   Fm1
......................................................................aagcaccaAgcuauuucaguuc....................  4      1   Fm1
......................................................................aagcaccagUcuauuucaguuc....................  10     1   Fm1
......................................................................aagcaccCggcuauuucaguuc....................  5      1   Fm1
......................................................................aagcacGaggcuauuucaguuc....................  6      1   Fm1
......................................................................aagcaccGggcuauuucaguuc....................  14     1   Fm1
......................................................................aagcaccaggcuauuuUaguuc....................  137    1   Fm1
......................................................................aagcaccagAcuauuucaguuc....................  7      1   Fm1
......................................................................Nagcaccaggcuauuucaguuc....................  26     1   Fm1
......................................................................aagcaccaggGuauuucaguuc....................  69     1   Fm1
......................................................................aagcaccaggAuauuucaguuc....................  125    1   Fm1
......................................................................Cagcaccaggcuauuucaguuc....................  3      1   Fm1
......................................................................aagcGccaggcuauuucaguuc....................  8      1   Fm1
......................................................................aaUcaccaggcuauuucaguuc....................  12     1   Fm1
......................................................................aagcaccaggcuauGucaguuc....................  3      1   Fm1
......................................................................aGgcaccaggcuauuucaguuc....................  15     1   Fm1
......................................................................aagcaccaggcuaGuucaguuc....................  6      1   Fm1
......................................................................aagcaccaggcuauuucagCuc....................  5      1   Fm1
......................................................................aagcaccaggcCauuucaguuc....................  6      1   Fm1
......................................................................aagcaccaggcuauuucaUuuc....................  9      1   Fm1
......................................................................aagcaccaggcuauuucaguuc....................  59669  0   Fm1
......................................................................aagcaccaggcuaAuucaguuc....................  7      1   Fm1
......................................................................aagcaUcaggcuauuucaguuc....................  23     1   Fm1
......................................................................aagcaccaCgcuauuucaguuc....................  3      1   Fm1
......................................................................aagcaccaggcAauuucaguuc....................  1      1   Fm1
......................................................................aagcCccaggcuauuucaguuc....................  5      1   Fm1
......................................................................aaAcaccaggcuauuucaguuc....................  7      1   Fm1
......................................................................aagcaccaggcuauuucUguuc....................  184    1   Fm1
......................................................................aagcaccaggcuauuCcaguuc....................  12     1   Fm1
......................................................................aagcaccaggcuauuucaguuU....................  415    1   Fm1
......................................................................aagAaccaggcuauuucaguuc....................  5      1   Fm1
......................................................................aagcaccaggcuauuucaguAc....................  2      1   Fm1
......................................................................aagcaccUggcuauuucaguuc....................  18     1   Fm1
......................................................................aagcaccaggcuauuuGaguuc....................  75     1   Fm1
......................................................................aagcacUaggcuauuucaguuc....................  22     1   Fm1
......................................................................aagcaccaggcuauuucGguuc....................  172    1   Fm1
......................................................................aagcaccagCcuauuucaguuc....................  3      1   Fm1
......................................................................aagcaccaggcuauuuAaguuc....................  224    1   Fm1
......................................................................aagcaccaggcuauuAcaguuc....................  5      1   Fm1
......................................................................Uagcaccaggcuauuucaguuc....................  34     1   Fm1
......................................................................aagGaccaggcuauuucaguuc....................  1      1   Fm1
......................................................................aagcaccaggUuauuucaguuc....................  54     1   Fm1
......................................................................aagcaNcaggcuauuucaguuc....................  11     1   Fm1
......................................................................aagcaccaggcuauuucaAuuc....................  3      1   Fm1
......................................................................aagcaccaggcuaCuucaguuc....................  10     1   Fm1
......................................................................aagcaccaggcuauuucaguuA....................  46     1   Fm1
......................................................................aagcaccaggcuauuucCguuc....................  174    1   Fm1
......................................................................aagcaccaggcGauuucaguuc....................  2      1   Fm1
```

```
                                  Star                                  Mature
agccagaacggagcugcugaaccuauccaguggacugggauuccuugugugcuguuugucauaucuuccaaagcaccaggcuauuucaguucucugguugggagaauuccac
......................................................................aagcaGcaggcuauuucaguuc....................    3     1    Fm1
......................................................................aaCcaccaggcuauuucaguuc....................    11    1    Fm1
......................................................................Gagcaccaggcuauuucaguuc....................    13    1    Fm1
......................................................................aagcaccaggcuauuucagAuc....................    4     1    Fm1
......................................................................aagcacNaggcuauuucaguuc....................    5     1    Fm1
......................................................................aUgcaccaggcuauuucaguuc....................    5     1    Fm1
......................................................................aagcaccaggcuauAucaguuc....................    5     1    Fm1
......................................................................aagcaccaggcuUuuucaguuc....................    4     1    Fm1
......................................................................aagcaccaggcuCuuucaguuc....................    3     1    Fm1
......................................................................aagcaccaggcuauuucaguCc....................    11    1    Fm1
......................................................................aagcaccaggcuauuucaCuuc....................    4     1    Fm1
......................................................................aagUaccaggcuauuucaguuc....................    2     1    Fm1
......................................................................aagcaAcaggcuauuucaguuc....................    9     1    Fm1
......................................................................aCgcaccaggcuauuucaguuc....................    2     1    Fm1
......................................................................aagcaccaUgcuauuucaguuc....................    11    1    Fm1
......................................................................aagcaccaggcuGuuucaguuc....................    2     1    Fm1
......................................................................aagcaccaggcuauuucagGuc....................    3     1    Fm1
......................................................................aagcUccaggcuauuucaguuc....................    3     1    Fm1
......................................................................aagcaccaggcuauuucaguucA...................    1764  1    Fm1
......................................................................aagcaccaggcuauuuGaguucu...................    2     1    Fm1
......................................................................aagcaccaUgcuauuucaguucu...................    1     1    Fm1
......................................................................aagcaccaggcuauuucaguucC...................    173   1    Fm1
......................................................................aagcaccaggGuauuucaguucu...................    4     1    Fm1
......................................................................aagcaccaggcuauuucGguucu...................    11    1    Fm1
......................................................................aagGaccaggcuauuucaguucu...................    3     1    Fm1
......................................................................aagcaccaggcuauuucagCucu...................    4     1    Fm1
......................................................................aagcUccaggcuauuucaguucu...................    1     1    Fm1
......................................................................aCgcaccaggcuauuucaguucu...................    1     1    Fm1
......................................................................aagcaccaggcuauuucUguucu...................    13    1    Fm1
......................................................................aagcaccaggcuauuucaguCcu...................    2     1    Fm1
......................................................................aagcaccaggcuauuucaguuUu...................    49    1    Fm1
......................................................................aagcaccaggcuauuucaguucu...................    4584  0    Fm1
......................................................................aagcaccaggcuauuucaguucG...................    19    1    Fm1
......................................................................aagcaccaggcuauuucaUuucu...................    1     1    Fm1
......................................................................aagcaccaggAuauuucaguucu...................    10    1    Fm1
......................................................................aagAaccaggcuauuucaguucu...................    1     1    Fm1
......................................................................Nagcaccaggcuauuucaguucu...................    3     1    Fm1
......................................................................aagcaccaggUuauuucaguucu...................    9     1    Fm1
......................................................................aagcaccaggcuauuuUaguucu...................    9     1    Fm1
......................................................................Gagcaccaggcuauuucaguucu...................    2     1    Fm1
......................................................................aagcaccagUcuauuucaguucu...................    1     1    Fm1
......................................................................aagcaccaggcuauuucaguuGu...................    6     1    Fm1
......................................................................aagcaUcaggcuauuucaguucu...................    2     1    Fm1
......................................................................aaAcaccaggcuauuucaguucu...................    1     1    Fm1
......................................................................aagcaccaggcuauuucagAucu...................    2     1    Fm1
......................................................................aagcaccaggcuauuucaguuAu...................    12    1    Fm1
......................................................................Uagcaccaggcuauuucaguucu...................    1     1    Fm1
......................................................................aagcaccaggcuauuucaAuucu...................    1     1    Fm1
......................................................................aagcaccUggcuauuucaguucu...................    1     1    Fm1
......................................................................aaUcaccaggcuauuucaguucu...................    2     1    Fm1
......................................................................aagcaccaggcuauGucaguucu...................    1     1    Fm1
......................................................................aagcaccaggcuauuuAaguucu...................    17    1    Fm1
......................................................................aagcaccaggcuauuucCguucu...................    16    1    Fm1
......................................................................aagcacAaggcuauuucaguucu...................    1     1    Fm1
......................................................................aagcaccaggcuaGuucaguucu...................    1     1    Fm1
......................................................................aagcaccaggcCauuucaguucu...................    2     1    Fm1
......................................................................aagcaccaggcuauuucaguucuA..................    18    1    Fm1
......................................................................aagcaccaggcuauuucaguucAc..................    2     1    Fm1
......................................................................aagcaccaggcuauuucaguucuc..................    1     0    Fm1
......................................................................aagcaccaggcuauuucaguucuU..................    239   1    Fm1
......................................................................aagcaccaggcuauuucaguucuUu.................    24    1    Fm1
.......................................................................agcaccaggcuauuucaguuc....................    20    0    Fm1
.......................................................................agcaccaggcuauuucagAuc....................    1     1    Fm1
.......................................................................agcaccaggcuauuucaguucu...................    1     0    Fm1
.........................................................................caccaggcuauuucaguucucu.................    3     0    Fm1
```
